# Editorial: When streptococci seize the opportunity: identifying how manipulation of host inflammatory signaling impacts pathogenesis in streptococcal infections

**DOI:** 10.3389/fcimb.2024.1364623

**Published:** 2024-01-15

**Authors:** Rebecca A. Flaherty, Shaun W. Lee, Shannon D. Manning

**Affiliations:** ^1^ Department of Biology and Health Science, Aquinas College, Grand Rapids, MI, United States; ^2^ Department of Biological Sciences, University of Notre Dame, Notre Dame, IN, United States; ^3^ Department of Microbiology and Molecular Genetics, Michigan State University, East Lansing, MI, United States

**Keywords:** streptococci, inflammation, immune response, infection, host signaling pathways

Many streptococcal pathogens such as *Streptococcus pyogenes, Streptococcus agalactiae*, and *Streptococcus pneumoniae* share the perplexing ability to live as relatively harmless human colonizers, while causing severe and potentially life-threatening illnesses in some cases. Severe diseases resulting from streptococcal infections are often marked by the induction of robust inflammatory responses in the infected host cells and surrounding tissues. The timing and type of inflammatory response can vary depending on factors such as pathovar type, which host cells are affected, and whether the host has certain genetic features or conditions that make them more susceptible to infection. In most cases, the activation of inflammatory signaling cascades benefits the host by alerting the immune system to the presence of microbial invaders. However, changes in the magnitude or type of inflammatory response can lead to pathogenic outcomes by causing extensive tissue damage at the primary infection site, promoting dissemination of the pathogen, and/or by triggering severe systemic outcomes such as septic shock.

The articles in this Research Topic reveal the manifold complexities of streptococcal-host interactions using a variety of approaches. Two featured articles address regulation and production of bacterial virulence factors from the perspective of the invading pathogen. Wahlenmayer and Hammers have reviewed the important contribution of small bacterial peptides to the complex relationships that exist between streptococcal pathogens and their hosts. Some of these bacterial peptides can directly interact with key host components to sabotage critical elements of the immune response. Some can even modulate the expression of host genes that aid in immune system regulation by acting as signaling mediators to other bacteria that possess immune-regulatory capabilities. Faozia et al. have investigated the bacterial mechanisms that regulate SpeB, a cysteine protease that is well-recognized as a key virulence factor of *Streptococcus pyogenes*. The authors have built on prior work that linked the loss of the c-di-AMP phosphodiesterase gene (*pde2)* with reduced expression of SpeB. They have identified both *dlt*X and the LiaFSR systems as suppressors of the SpeB-null phenotype in mutants that lack *pde2. dlt*X is a member of the *dlt* operon, which is responsible for the incorporation of D-alanine into lipoteichoic acids. Because its loss led to restored SpeB production in the *Δpde2* mutant strain, the authors have concluded that D-alanylation is crucial for the suppressor phenotype. Similarly, they have demonstrated that mutations in the LiaFSR system, which detects and responds to cell envelope stress, can derepress SpeB via SpxA2 regulation, though these two SpeB regulatory mechanisms do not appear to be linked.

Other articles in the Research Topic explore cell signaling and immune responses to these bacterial pathogens from the host perspective. De-Leon-Lopez et al. have used a cell culture-based approach to explore the role of the PI3K-Akt pathway in several stages of Group B *Streptococcus* (GBS) pathogenesis in macrophages. The authors have demonstrated that activation of PI3K-Akt signaling by GBS promotes uptake of GBS by macrophages via actin cytoskeletal rearrangements, survival of intracellular GBS, activation of the NFκB pathway, and survival of the GBS-infected macrophages. Notably, strains associated with more severe pregnancy and neonatal complications, such as capsule type III and sequence type (ST)-17 strains, exhibited an enhanced ability to activate the PI3K-Akt pathway, which may contribute to the heightened virulence of these strains.

The study by Manuel et al. also focuses on key host immune responses to GBS, but does so at the organismal level using pregnant macaques. Use of this complex model has allowed the researchers to examine GBS-induced inflammatory factors that contribute to preterm birth. In this study, the authors have detected protein changes involved in immune system regulation at the maternal-fetal interface over the course of infection using multiple strains of GBS. Changes in immune checkpoint protein expression in the amnion, chorion, and maternal decidua have been systematically examined, and interestingly, numerous stimulatory factors as well as a variety of inhibitory factors have been found to be upregulated in these tissues. This study highlights the complex factors involved in achieving an effective immune response to GBS. The authors have concluded that a reduction in immune system activation may benefit the host by restricting immune-driven pathologies but may simultaneously risk benefiting the pathogen by limiting immune surveillance.

The article by Ni et al. also showcases the threat posed by an inappropriately tailored immune response at the organismal level. This study has specifically evaluated the importance of the major histocompatibility complex (MHC) in the interplay between the zoonotic pathogen *Streptococcus suis* and the host in a transgenic mouse model. Humanized transgenic HLA-A11/DR1 genotype mice have been compared to wild-type murine H2 mice at multiple time points following *S. suis* infection. The transgenic mice have been shown to display elevated levels of pro-inflammatory cytokines, more pronounced tissue destruction, enhanced recruitment of granulocytes, and a diminished ability to resolve the infection when compared with their wild-type counterparts. Lower levels of the anti-inflammatory cytokine IL-10 and production of fewer regulatory T cells in the humanized mice have been cited as key factors contributing to the prolonged inflammation in the humanized mice.

Altogether, this Research Topic highlights the complex relationship between host states and virulence factors that are at play during host interactions with different types of streptococcal species. The studies featured here will allow us to better connect the observed changes in specific host mediators to the corresponding stages of disease that they impact, such as colonization, immune cell activation or recruitment, and tissue destruction. Such connections are a key step toward the development of effective diagnostic and therapeutic strategies that can temper the devastating effects of streptococcal pathogens. There is still much to be learned in this field, and the perplexity of why these intriguing pathogens can harmlessly colonize yet cause severe disease in many cases remains to be fully elucidated. We would like to extend our sincere gratitude to the authors who contributed their excellent work to this Research Topic as well as to the reviewers for providing their time and insightful comments.

## Author contributions

RF: Writing – original draft. SL: Writing – review & editing. SM: Writing – review & editing.

